# Long Non-coding Wilms Tumor 1 Antisense RNA in the Development and Progression of Malignant Tumors

**DOI:** 10.3389/fonc.2020.00035

**Published:** 2020-02-14

**Authors:** Ye Zhang, Lin-Jun Fan, Yi Zhang, Jun Jiang, Xiao-Wei Qi

**Affiliations:** Department of Breast and Thyroid Surgery, Southwest Hospital, Third Military Medical University (Army Medical University), Chongqing, China

**Keywords:** Wilms tumor 1 antisense RNA, tumor, long non-coding RNA, expression, function

## Abstract

A growing number of studies have shown that long non-coding RNAs (lncRNAs) play an important role in tumor development and progression and are key molecules affecting tumor progression. The lncRNA Wilms tumor 1 antisense RNA (WT1-AS) is specifically expressed in various malignant tumors. In particular, WT1-AS expression is upregulated in colon cancer and breast cancer but is significantly downregulated in cervical cancer, liver cancer, and kidney cancer. The level of WT1-AS expression is closely related to the size, stage, and patient survival rate of these cancers. In this article, we review the modes of action, expression, function, and mechanisms of WT1-AS in different tumors to provide new targets for tumor diagnosis and treatment.

## Introduction

Non-coding RNAs (ncRNAs) are a class of non-protein-coding RNAs that mainly include two kinds of RNA: (1) small ncRNAs (sncRNAs) with a length of 21–200 nucleotides such as microRNAs (21–25 nucleotides), transfer RNAs (tRNAs), and small interfering RNAs (siRNA); and (2) long ncRNAs (lncRNAs) with a length of over 200 nucleotides (1, 2). Such ncRNAs play different roles in cells. For example, tRNAs are responsible for carrying and transferring amino acids and are involved in protein translation; sncRNAs are widely used for RNA interference (RNAi), which may act as primers to synthesize double-stranded RNAs (dsRNAs), and then such dsRNAs could serve to amplify siRNA response and allow spreading of RNAi silencing (2).

With the increasing understanding of ncRNAs, multi-faceted, comprehensive, and in-depth studies on their functions, mechanisms of action, and their interactive networks are required to determine their roles under specific conditions, and gain further understanding of various physiological, and pathological processes.

LncRNAs have transcripts of >200 nucleotides (nt) in length. Although lncRNAs barely encode proteins, they can regulate gene expression and participate in various molecular biological processes through mechanisms such as RNAi, gene co-suppression, gene silencing, gene imprinting, and DNA demethylation (2).

LncRNAs are highly diverse and complex endogenous ncRNA molecules involved in the regulation of many biological processes (3, 4). Wilusz et al. and Wang et al. reviewed the function of lncRNAs and proposed that their action can be divided into four modes. (1) Signal: lncRNAs can be activated under specific conditions such as specific expression, DNA damage, and cold condition, and can participate in the regulation of downstream gene expression. (2) Molecular decoy: lncRNAs can form a complex regulatory network directly with proteins or other RNAs once transcribed and can act as molecular blocks to block the translation of target genes. (3) Guide: lncRNAs can bind to proteins and localize the latter to specific DNA sequences to regulate DNA transcription. The guide role of lncRNAs can be further divided into *cis*-guiding and *trans*-guiding. (4) Scaffold: Protein complexes can bind to lncRNAs and then, through the lncRNAs, can bind to chromatins and DNAs. This enables the regulation of different signaling pathways and the exchange and integration of information (5, 6). Additionally, lncRNAs can also directly participate in protein translation and modification. For example, the antisense RNA of ubiquitin carboxy-terminal hydrolase L1 (*Uchl1*) can participate in the translation and maintain the stability of *Uchl1* mRNA (7). Moreover, overexpressed in colon carcinoma 1 (OCC1) can promote ubiquitination by recruiting the E3 ligase β-TrCP1 and stabilizing its binding to Hu antigen R (HUR) protein (8). Interestingly, Anderson et al. reported that the lncRNA LINC00948 could serve as templates for the translation of functional micropeptides myoregulin (MLN), and MLN was an essential regulator of skeletal muscle activities (9). Similarly, Lu et al. reported the same function of lncRNA and detected 308 lncRNA-encoded new peptides according to shotgun proteomics (10). Those results indicate that lncRNAs are complex and versatile regulators that may be involved in many biological processes. Therefore, great challenges lie in the understanding of the molecular biology of lncRNAs and their uncharted interactions in human disease.

Wilms tumor 1 (*WT1*) is a transcription factor that plays an important role in genitourinary system development and an inhibitory role in the development and progression of Wilms tumor. In addition, *WT1* is widely expressed during fetal spleen, spinal cord, and brain development, suggesting its involvement in the development of these organs (11, 12). The WT1 antisense RNA (WT1-AS) originates from the intron region of WT1 (13). Its expression is regulated by methylation and abnormal splicing and is closely associated with the development and progression of various tumors (14). The function of WT1-AS is highly tissue- and cell-specific and may play distinct roles in different tumors. In-depth research on the roles of WT1-AS in different tumors and its possible mechanisms of action is of great value in cancer diagnosis and treatment. This article provides an overview of the modes of action, expression, and function of WT1-AS in different tumors.

## The Modes of Action of Wilms Tumor 1 Antisense RNA in Malignant Tumors

As the antisense RNA of *WT1*, WT1-AS primarily acts as a signal and molecular decoy in tumors. Similar to *WT1*, WT1-AS is highly expressed in embryonic kidneys and is highly correlated with *WT1* expression. A subsequent study has demonstrated that WT1-AS can bind to *WT1* mRNA and regulate WT1 protein expression through RNA–RNA interactions (13). Current research shows that the primary modes of action of WT1-AS are signal and molecular decoy. In Wilms tumor and acute myeloid leukemia (AML), a similar mechanism related to WT1-AS–WT1 interaction could regulate the expression of WT1 protein (15). In liver cancer cells, WT1-AS can bind directly to the TATA region of the *WT1* promoter to downregulate *WT1* gene expression (16). Moreover, WT1-AS can bind to microRNAs such as miR-203a-5p and miR-330-5p as a molecular decoy and can inhibit the translation of downstream genes, including *TP53* (tumor protein p53) and *FOXN2* (forkhead box N2) (17, 18), thereby regulating the biological behaviors of tumor cells. Recent studies have found that WT1-AS plays important roles in many tumors, but its role and *WT1* and *TP53* gene expression regulation vary significantly between tumors. Therefore, understanding the specific roles and mechanisms of action of WT1-AS in different tumors can shed new light on comprehensive understanding of the dynamic changes in tumor development and progression and on the search for therapeutic strategies targeting lncRNAs.

## Expression Level of Wilms Tumor 1 Antisense RNA in Malignant Tumor Cells

**Figure 1:**
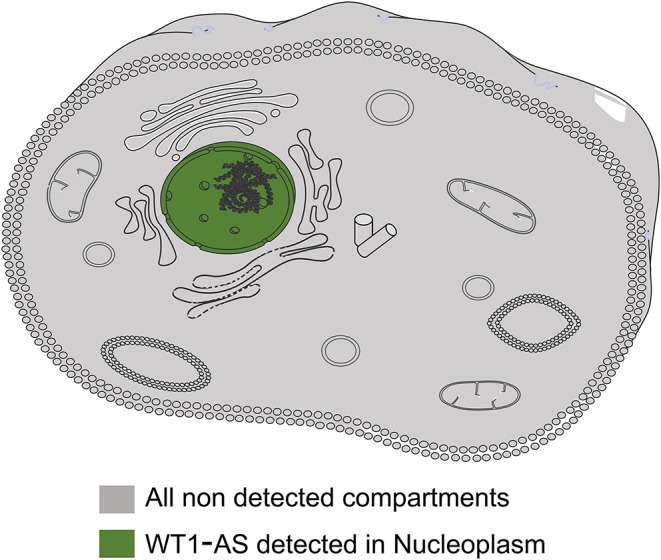
Representative image of cellular localization of Wilms tumor 1 antisense RNA (WT1-AS). The information of WT1-AS cellular localization was derived from RNALocate and lncATLAS; the information provided evidence that WT1-AS is mainly located in the nucleus.

WT1 is an important transcription factor in various tumors and is mainly located in the nucleus. To identify the subcellular localization of WT1-AS, a search through the RNA localization databases was performed (RNALocate: http://www.rna-society.org/rnalocate/index.html and lncATLAS: http://lncatlas.crg.eu/), which showed that WT1-AS is mainly located in the nucleus, which is similar to WT1 (Figure 1).

Numerous studies have shown that WT1-AS expression in tumors is tissue-specific and closely related to tumor development and progression, which is summarized in Table 1. Using bioinformatics, Zhang et al. found that WT1-AS expression was upregulated in colon cancer tissue compared with paracancerous tissues, and patients with colorectal cancer with high WT1-AS expression had poorer prognosis (20). Similarly, Sun et al. screened The Cancer Genome Atlas (TCGA) database for lncRNAs with predictive value for breast cancer prognosis and found that WT1-AS was upregulated in breast cancer. Patients with breast cancer with high WT1-AS expression also had poorer prognosis (19). In addition, others have found that WT1-AS expression is upregulated in AML and Wilms tumor (15, 21). WT1-AS is highly expressed in the aforementioned malignant tumors, and its high expression is significantly associated with late staging and shortened overall survival time. Knocking down WT1-AS expression can significantly reduce tumor cell proliferation and migration.

**Table 1:** The association between WT1-AS and the prognosis of patients with malignant tumors.

**Tumor type**	**Expression pattern**	**Relationship with clinical parameters and prognosis**	**References**
Breast cancer	Upregulated	Patients with high WT1-AS expression had poorer prognosis.	(19)
Colorectal cancer	Upregulated	Patients with high WT1-AS expression had poorer prognosis.	(20)
AML	Upregulated		(15, 21)
Wilms tumor	Upregulated		(15, 21)
Gastric cancer	Downregulated	Low expression was related to clinicopathological parameters such as late staging of the tumor and high degree of infiltration.	(22)
Cervical cancer	Downregulated	Patients with low expression had higher FIGO stage, were more susceptible to lymph node metastasis, and had poorer prognosis.	(17, 18)
Liver cancer	Downregulated	Patients with low WT1-AS expression had lower 5 years survival rates.	(16)
Kidney cancer	Downregulated	WT1-AS expression was an independent predictor of the prognosis of clear cell renal cell carcinoma, and patients with high WT1-AS expression had poorer prognosis.	(23, 24)
Ovarian cancer	Upregulated	Patients with low WT1-AS expression had poor prognosis.	(25–28)

*WT1-AS, Wilms tumor 1 antisense RNA; AML, acute myeloid leukemia; FIGO, International Federation of Gynecology and Obstetrics*.

However, WT1-AS is significantly downregulated in tumors such as gastric cancer, cervical cancer, liver cancer, and kidney cancer; and its biological functions are also quite different. WT1-AS expression is downregulated in gastric cancer tissue compared with that in normal gastric tissue, and its low expression is related to clinicopathological parameters such as late staging of the tumor and high degree of tumor invasion (22). Dai et al. found that WT1-AS expression was downregulated in cervical cancer tissue and that patients with low WT1-AS expression had higher International Federation of Gynecology and Obstetrics (FIGO) stage and were more susceptible to lymph node metastasis (18). Similarly, Cui et al. demonstrated that patients with cervical cancer with low WT1-AS expression had poorer prognosis (17). Lv et al. found lower WT1-AS expression in liver cancer tissue than in cancer-adjacent tissue, and patients with low WT1-AS expression had lower 5 years survival rates (16). Yang et al. compared the lncRNA expression profiles of clear cell renal cell carcinoma (ccRCC) and normal tissue. They found lower WT1-AS expression in ccRCC than in normal tissue, and WT1-AS expression level was significantly correlated with prognosis (23). Moreover, a subsequent study revealed that WT1-AS expression can be used as an independent predictor of ccRCC prognosis and that patients with high WT1-AS expression had poorer prognosis (24).

Interestingly, WT1-AS is differentially expressed in different histological subtypes of ovarian cancer: WT1-AS expression is higher in ovarian cancer tissue than in normal tissue (25). However, CpG island methylation of the *WT1*/*WT1*-*AS* promoter is higher in ovarian clear cell adenocarcinoma, compared with ovarian serous adenocarcinoma, resulting in the differential expression of WT1-AS between these two tumor types. This may also be the reason why clear cell adenocarcinoma has poorer prognosis than serous adenocarcinoma (26). Similarly, Niskakoski et al. analyzed the differential expression of lncRNAs in various histological subtypes of ovarian cancer and found that non-serous ovarian cancer had a greater degree of epigenetic WT1-AS inactivation than ovarian serous adenocarcinoma (27). In another study, Wang et al. analyzed the competing endogenous RNA (ceRNA) network and found that patients with recurrent ovarian cancer with low WT1-AS expression had poorer prognosis (28).

The above studies demonstrate that WT1-AS expression level is significantly tissue- and cell-specific in different tumors. WT1-AS plays a cancer-promoting role in some tumors but a tumor-suppressing role in others, which may be attributable to the tissue-specific expression of lncRNAs and their complex regulatory network. Therefore, further understanding of the specific roles and mechanisms of action of WT1-AS in different tumors and exploration of the causes of differential WT1-AS expression between tumors is important.

## Specific Roles and Mechanisms of Action of Wilms Tumor 1 Antisense RNA in Tumors

### Wilms Tumor 1 Antisense RNA and Wilms Tumor 1

Although the *WT1* gene plays a tumor-suppressing role in nephroblastoma, it is highly expressed and plays an oncogenic role in breast cancer, lung cancer, and colorectal cancer (29). WT1 has a wide range of biological effects. It not only recognizes and binds to specific target DNAs as a transcription factor and regulates gene transcription but also binds to various growth regulators and the corresponding receptors, thereby playing an important role in cell signal transduction (30). WT1-AS can regulate WT1 protein expression, but its regulatory effects and mechanisms of action vary between tumors. Dallosso et al. found that AML and Wilms tumor had high *WT1* mutation rates and high *WT1*-*AS* expression levels (15). WT1-AS may bind to *WT1* mRNA to form a heteroduplex, thereby regulating WT1 protein expression (13). However, how such a heteroduplex is involved in *WT1* expression remains unclear; therefore, the exact mechanism remains to be elucidated. Lv et al. analyzed the correlation between WT1-AS and WT1 in liver cancer and found that WT1-AS was negatively correlated with *WT1* expression. WT1-AS can bind to the TATA region of the WT1 promoter to inhibit WT1 transcription (16).

**Figure 2:**
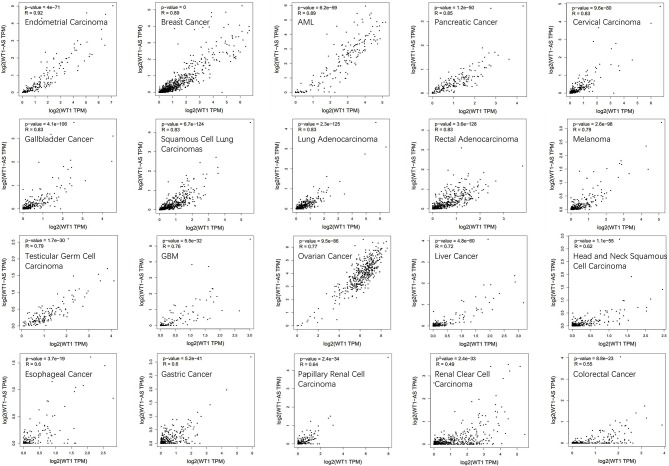
Correlation analysis of Wilms tumor 1 antisense RNA (WT1-AS) and Wilms tumor 1 (*WT1*) mRNA. Data are presented as log scaled, and the correlation analysis was derived from Gene Expression Profiling Interactive Analysis (GEPIA). The correlation coefficient in different cancer types was calculated using the Spearman test. Representative results from 20 common cancers are shown according to their own correlation coefficient. *R*, correlation co-efficient.

To determine the correlation between *WT1* mRNA and WT1-AS expression, we performed correlation analysis using Gene Expression Profiling Interactive Analysis (GEPIA; http://gepia.cancer-pku.cn/, an online database based on TCGA), and we found that *WT1* expression is significantly positively correlated with WT1-AS expression in most tumors, which is consistent with the findings of previous studies (including liver cancer) but not with the results of Lv et al. (16) (Figure 2). We speculate that this difference is attributable to the fact that the cases collected for the present study were mainly Chinese patients with liver cancer admitted to Nanjing Medical University, whereas the patients included in the TCGA database were mainly from the United States and Europe. Different from those in Asian countries such as China and Japan, where liver cancer is primarily caused by viral hepatitis, cases of liver cancer in the United States and Europe are primarily caused by chronic liver disease resulting from obesity and alcoholism (31, 32). This suggests that WT1-AS and WT1 may have different modes of action under different pathogenic conditions.

The feedback regulatory network of WT1-AS and WT1 may be the key to the different effects of WT1-AS on tumors, but the specific regulatory mechanism remains unclear but is possibly attributable to the methylation level of WT1 sense and antisense strands, histone methylation, and acetylation modifications. At the same time, *WT1* and *WT1*-*AS* mutations and splice variants may differ between tumors; therefore, these genes may play different roles in various tumors. In-depth study of the WT1–WT1-AS regulatory network in tumors may provide more possibilities for further understanding of the molecular biological characteristics of tumors and for developing new targeted drugs.

### Wilms Tumor 1 Antisense RNA and Tumor Cell Proliferation and Apoptosis

The basic biological characteristics of tumor cells are infinite proliferative potential and resistance to death (33). WT1-AS is closely associated with tumor cell proliferation and apoptosis, and its effect on proliferation and apoptosis differs between tumors. Du et al. overexpressed WT1-AS in gastric cancer cell lines and observed slowed cell proliferation and more G1/G0-phase cells. *In vivo* experiments demonstrated that WT1-AS overexpression inhibited tumor formation in mice. The authors also reported that WT1-AS overexpression in gastric cancer cells reduced extracellular signal-regulated kinase (ERK) protein phosphorylation, thereby inhibiting gastric cancer cell proliferation (22). Similar findings have also been observed in cervical cancer (17, 18).

Silencing WT1-AS in ovarian serous adenocarcinoma cell lines can inhibit tumor cell proliferation and downregulate the expression of various oncogenes. In contrast, WT1-AS overexpression can promote tumor cell proliferation (27). Lv et al. found that WT1-AS can downregulate *WT1* expression in liver cancer cells, thereby blocking the JAK/STAT3 (signal transducer and activator of transcription 3) signaling pathway and promoting liver cancer cell apoptosis (16).

The above studies demonstrate that WT1-AS is closely associated with many biological behaviors such as tumor cell proliferation, cell cycle arrest, and resistance to cell death, and that WT1-AS plays different roles in various tumors. Elucidating the regulatory mechanism of WT1-AS in tumor cell proliferation and apoptosis would provide insight in to the development of corresponding diagnostic and treatment strategies to better target and regulate tumor growth.

### Wilms Tumor 1 Antisense RNA and Tumor Invasion and Metastasis

The major features of malignant epithelial tumors are invasion and metastasis. They are not only the focus and obstacle of tumor treatment but also complex processes influenced by various regulatory factors, which determine the prognosis of patients with cancer (33). Cui et al. reported that WT1-AS knockdown in the SiHa and CaSKi cervical cancer cell lines increased the invasive and migration abilities of these tumor cells, whereas WT1-AS overexpression attenuated their invasive and migration abilities (17); Dai et al. reported similar results (18). Du et al. found that patients with gastric cancer with low WT1-AS expression were more likely to have local invasion and distant metastasis, whereas WT1-AS overexpression in the HGC7901 and HS-746T gastric cancer cell lines attenuated cell invasion and migration (22). The results of these investigational studies are consistent with the correlation between WT1-AS expression and the clinicopathological parameters of patients with cervical cancer or gastric cancer. However, the specific regulatory mechanism of WT1-AS in the invasion and metastasis of these cancers is still unclear and needs further exploration. Additionally, the regulatory effect of WT1-AS on tumor cell invasion and migration in other cancers remains to be explored further.

**Figure 3:**
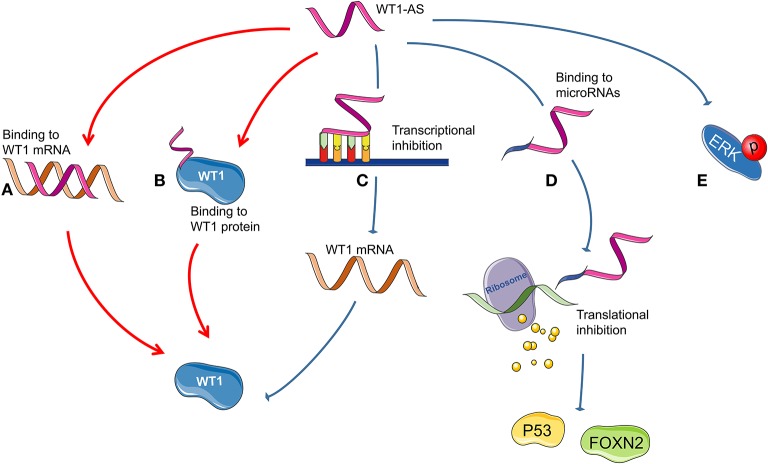
Possible mechanism of Wilms tumor 1 antisense RNA (WT1-AS) functioning. **(A)** A heteroduplex formed by the combination of WT1-AS and Wilms tumor 1 (*WT1*) mRNA could regulate the expression of WT1 protein. **(B)** WT1-AS can bind to WT1 protein to regulate the abundance of WT1 protein. **(C)** WT1-AS can directly bind to the TATA region of the *WT1* promoter, inhibit *WT1* transcription, and downregulate WT1 protein expression, thereby inhibiting the JAK/STAT3 signaling pathway. **(D)** WT1-AS can bind as a molecular decoy to miR-203a-5p and miR-330-5p and can inhibit the translation of downstream genes, including *TP53* and *FOXN2*. **(E)** WT1-AS can reduce extracellular signal-regulated kinase (ERK) phosphorylation.

Figure 3 summarizes all the reported mechanisms of WT1-AS in malignant tumors.

## Conclusions

As research on the function and mechanisms of action of lncRNAs advances, their role in tumor development and progression has gradually become clearer, and their significance in cancer diagnosis and treatment has attracted increasing research attention. To date, the research progress in WT1-AS appears to clarify that it has some interesting features. Current research indicates that WT1-AS is specifically expressed in malignant tumors, and its expression level is closely related to clinicopathological parameters such as tumor size, tumor-node-metastasis (TNM) stage, and survival, indicating that it plays an important role in such malignant tumors and may serve as a new target for tumor diagnosis and treatment. It is worth noting that WT1-AS plays distinct roles in different tumors, wherein it may be tumor suppressive in some while being cancer promoting in others. Therefore, the specific roles of WT1-AS in different tumors require further validation. As for the antisense RNA of WT1, future progress will be made with the development of new RNA detection technologies to indicate the interaction between WT1 and WT1-AS and its intrinsic mechanisms in different tumors. In addition, the specific regulatory mechanisms of WT1-AS in tumor development and progression remain unclear and require further investigation.

## Author Contributions

YeZ contributed to the drafting of the manuscript. L-JF and YiZ contributed to the literature search. JJ modified the language. X-WQ contributed to the conception or design of the work. All authors have read and approved the final manuscript.

### Conflict of Interest

The authors declare that the research was conducted in the absence of any commercial or financial relationships that could be construed as a potential conflict of interest.
